# Family Experiences of Integrated Care for Children With Medical Complexity: A Scoping Review

**DOI:** 10.1111/cch.70091

**Published:** 2025-05-07

**Authors:** Yvonne Zurynski, Karen Hutchinson, Yilin Kang, Maryam Vizheh, Anneliese de Groot

**Affiliations:** ^1^ Australian Institute of Health Innovation Macquarie University Macquarie Park New South Wales Australia; ^2^ Faculty of Medicine, Health and Human Sciences Macquarie University Macquarie Park New South Wales Australia; ^3^ Central Coast Local Health District NSW Health Gosford New South Wales Australia; ^4^ Central Coast Research Institute Gosford New South Wales Australia; ^5^ Northern NSW Local Health District NSW Health Lismore Australia

**Keywords:** care experiences, children with medical complexity, family‐centred care, integrated care, models of care

## Abstract

**Background:**

Children with medical complexity (CMC) frequently access multiple healthcare services across often fragmented systems. Paediatric integrated care models (PICMs) support health care coordination, but little is known about experiences and perceived benefits and barriers among CMC, parents or carers while accessing PICMs. This review addresses these knowledge gaps by synthesising current published evidence.

**Methods:**

A scoping literature review based on searches of four databases: Medline, Embase, Scopus and CINAHL (2015–2024). Articles reporting on experiences of accessing PICMs by CMC aged < 19 years, their parents or carers were included. Data were extracted and thematically synthesised to describe experiences and perceived benefits and barriers.

**Results:**

The seven included papers reported on the experiences of parents (mostly mothers, 89%); only one paper included the views of CMC and siblings. All seven papers described the benefits of PICMs, including greater attention to individualised needs, smoother system navigation facilitated by care coordinators and improved communication and information sharing among care teams. Four papers reported barriers including limited understanding among parents and carers of care coordinator roles and processes and pathways of PICMs. Systemic barriers limited medical records sharing across providers and settings, and in two studies, parents raised this as a risk for care quality and safety for their CMC. Other systemic barriers identified by parents included a lack of stable funding for new models of care and difficulties linking PICMs with primary care, social care and education sectors.

**Conclusions:**

The evidence on experiences, benefits and barriers of PICMs among CMC, families and carers is scarce, and the voices of CMC are largely absent. The greater involvement of CMC, their parents and carers in the design and ongoing evaluation of PICMs should be a priority to improve family‐centred integrated care for CMC.

**Summary**

- Currently, there is scarce evidence in the peer‐reviewed literature about families' experiences and perspectives of engaging with paediatric integrated care models for CMC.
- Most studies of parents and carers who engage with paediatric integrated care models report experiencing benefits in terms of better satisfaction with care and smoother navigation of care services.
- Research evaluating the experiences of paediatric integrated care models for CMC has focused predominantly on mothers. Future research should include the voices of other family members and of CMC themselves to gain a more holistic understanding of experiences, benefits and barriers.
- Information for families and children is needed when they first engage with paediatric integrated care models to ensure they understand the scope, processes and pathways.
- CMC and their parents or carers must be engaged in the co‐design, implementation and evaluation of future models of integrated care to ensure these models are fit for purpose.

## 1 Introduction

Children with medical complexity (CMC) live with chronic developmental, physical and/or behavioural conditions. They require frequent access to health services across multiple care providers and often use medical and adaptive technology to support activities of daily living (Altman et al. 2018; Cohen et al. 2011; Millar et al. 2024). The four domains of medical complexity—‘diagnostic conditions, functional limitations, healthcare use, and family‐identified needs’ proposed by Cohen et al. (2011)—have recently been revised by Millar et al. (2024) to define consensus‐based standardised criteria for each domain. Importantly, Millar et al. (2024) include the social determinants of health as integral to the concept of medical complexity. Families living with CMC face many challenges navigating fragmented health systems that are predominantly based on episodic care and often fail to meet the unique, interdisciplinary and continuous care needs of CMC (Altman et al. 2018). Common challenges highlighted by parents include juggling family and work commitments while accessing multiple healthcare services and providers for their child, and this is often compounded by financial, social and emotional concerns (Altman et al. 2018; Cohen et al. 2011; Cohen and Coller 2020; Millar et al. 2024). Parents describe ineffective communication and poor care coordination among different healthcare teams and providers, especially when numerous specialists are involved in care (Altman et al. 2018; Breen et al. 2018; Lingam et al. 2023). This lack of care integration and coordination can result in missed or duplicated care and out‐of‐pocket family expenses associated with additional travel and time off work to attend appointments, which is additionally challenging for families living in rural locations (Altman et al. 2018; Lingam et al. 2023).

Paediatric integrated care models (PICMs) provide a potential solution for more timely, equitable, family‐centred, coordinated care that is valued by children and families and healthcare providers who deliver care (Breen et al. 2018; Wolfe et al. 2016; Wolfe et al. 2020). Integrated care has been variously defined, but continuity and coordination of care and a person‐centred approach are key components (Goodwin et al. 2022; Lennox‐Chhugani 2021). The International Foundation for Integrated Care defines integrated care as ‘joined‐up, easy to navigate care that addresses the outcomes that matter to people in their life and the communities in which they live’ (Lennox‐Chhugani et al. 2022). Aligning with the Quintuple Aim in healthcare (Nundy et al. 2022), PICMs have been shown to improve health outcomes, reduce costs, increase provider and patient satisfaction and address equity barriers (Bodenheimer and Sinsky 2014; Breen et al. 2018; Sikka et al. 2015; Yu et al. 2020). PICMs for CMC offer a solution to improve the experiences of CMC and their parents or carers by creating healthcare systems that are more responsive, connected and child and family‐focused (Altman et al. 2018; Breen et al. 2018). In this paper, a carer is defined as a family member other than a parent or another individual who takes the primary responsibility of the day‐to‐day care, support and advocacy of CMC (Hoover et al. 2022).

The evidence on the benefits of PICMs as perceived by CMC, their parents or carers is currently limited. Therefore, a scoping literature review was conducted to:

1. Describe the PICMs and their components as reported in studies that include the perspectives of CMC, parents or carers, and
2. Synthesise experiences, perspectives, benefits and barriers of CMCs, parents or carers while accessing PICMs.

## 2 Methods

### 2.1 Study Design

A scoping literature review was conducted, guided by the Preferred Reporting Items for Systematic Review and Meta‐Analysis Scoping Review (PRISMA‐ScR) statement and recommendations (Tricco et al. 2018). Scoping reviews enable the systematic mapping of knowledge and knowledge gaps in an emerging area of research where evidence based on randomised trials may be limited and support the synthesis of evidence from qualitative, quantitative and mixed methods studies. In addition, this study was guided by the scoping review methodology described by Levac et al. (2010). According to PRISMA‐ScR, protocol registration is not required for scoping reviews (Tricco et al. 2018), and no protocol for this study is registered or published. The completed PRISMA‐ScR Checklist is provided in Appendix S1.

### 2.2 Search Strategy and Study Selection Criteria

The search strategy was developed by members of the team in liaison with a medical librarian. The search was guided by the SPIDER framework for qualitative evidence searching and synthesis (Sample, Phenomenon of Interest, Design, Evaluation, Research Type) (Cooke et al. 2012). Initial exploratory searches were performed in 2020 by a student as part of their Doctor of Medicine Research Project requirements. The comprehensive search was undertaken in June 2022 and updated in November 2023 and May 2024 using four academic databases: Medline, Embase, Scopus and CINAHL. An example of the search strategy can be found in Table S1. The inclusion and exclusion criteria were aligned with the SPIDER framework (Cooke et al. 2012), (Table 1). To be included, studies had to report the experiences and perspectives of CMC, their parents or carers. The search strategy was limited to high‐income countries (Organisation of Economic Cooperation and Development (OECD) 2024) to enable meaningful synthesis and interpretation of results reported from countries with healthcare systems that share similar features such as access to advanced medical technologies and capacity for developing PICMs. As the implementation of PICMs and the study of their benefits is a recent and rapidly developing area of research, we conducted our search from 2015 to 2024. Due to resource constraints and the known methodological limitations of reliably searching and identifying relevant grey literature (Lefebvre et al. 2024), grey literature was excluded.

**TABLE 1:** Inclusion and exclusion criteria guided by the SPIDER framework (Cooke et al. 2012).

SPIDER domain	Inclusion criteria	Exclusion criteria
Sample	CMC aged under 19 years or the parents, carers or family members of CMC	CMC older than 19 years or practitioners or health professionals rather than children, parents, family members or carers
Phenomenon of Interest	Study reports on the experiences of accessing implemented PICM	Study does not report on an implemented PICM or care coordination Study reports on the design of a model of PICM or care coordination that has not been implemented
Design	Observational, descriptive single‐point, randomised controlled trials or pre–post evaluation studies	Study used methods that did not collect data describing CMC, family or carer experiences
Evaluation	Study reports on the experiences and perspectives of CMC or their parents, family members or carers	Study does not report on the experiences or perspectives of CMC or their family members or carers
Research type	Qualitative, quantitative and mixed methods studies Empirical studies published as peer‐reviewed papers	Opinions; letters; posters; conference proceedings; thesis dissertations; systematic reviews; protocols
Other selection criteria		
Publication date	Published after 1 January 2015 and before 12 January 2023	Published before 1 January 2015 or after 12 January 2023
Language	English	All other languages
Setting	Any setting where paediatric integrated healthcare is delivered (primary community and hospital sectors)	NA
Country	High‐income OECD countries	Low or middle‐income OECD countries

Abbreviations: CMC, children with medical complexity; OECD, Organisation for Economic Cooperation and Development (Organisation of Economic Cooperation and Development (OECD) 2024); PICM, paediatric integrated care models; SPIDER, Sample, Phenomenon of Interest, Design, Evaluation, Research Type.

### 2.3 Study Selection Process

Search results were downloaded into EndNote V20, and duplicates were removed. Titles and abstracts were then independently screened by two researchers according to selection criteria, with two authors overseeing the process and clarifying the application of selection criteria during regular meetings. Studies that met the criteria after title and abstract screening underwent full‐text review by two authors. During regular team meetings, all authors discussed the decisions, resolved any uncertainties and agreed on the final set of included studies. The reference lists of included articles were scanned initially by multiple authors to identify any additional articles that might have been missed in the database search.

### 2.4 Data Extraction

A purpose‐designed Excel spreadsheet was developed and piloted for data extraction. The following details were extracted: study characteristics (authors, year of publication, setting and design), population characteristics (sample size, age of CMC and type of medical complexity), PICM details and key components, experiences of CMC, parents, family members or carers, including perceived benefits and barriers while accessing the PICM. In papers that included data collected from multiple groups, only data provided by CMC, parents, family members or carers were extracted for analysis in keeping with the aims of this study. Data extraction occurred in tandem with the full‐text review. All data extraction was cross‐checked by two team members to ensure accuracy and completeness.

### 2.5 Quality Assessment

The methodological quality of included studies was assessed using Hawker's Quality Assessment Tool for diverse study types and designs (Hawker et al. 2002). Two authors conducted the quality assessment, which was checked by a senior member of the team. In keeping with the scoping review methodology, quality assessment was conducted to describe the methodological quality of included studies, rather than to exclude studies of poor quality from the review.

### 2.6 Data Analysis and Synthesis

A modified approach described by Levac et al. was applied, avoiding re‐interpretation of included studies, staying true to the findings reported by the original authors (Levac et al. 2010). The extracted information about PICM details and components was tabulated and described using simple frequencies and narrative descriptions. A modified approach to analysis as described by Mays et al. guided the narrative synthesis (Mays et al. 2005) to identify common themes emerging from the extracted data. Two authors conducted this analysis, and the synthesis was discussed and refined by the whole team. Emerging themes on key benefits and barriers experienced by CMC, parents or carers accessing PICMs were identified and aggregated across the included studies, tabulated and narratively described.

## 3 Results

**FIGURE 1:**
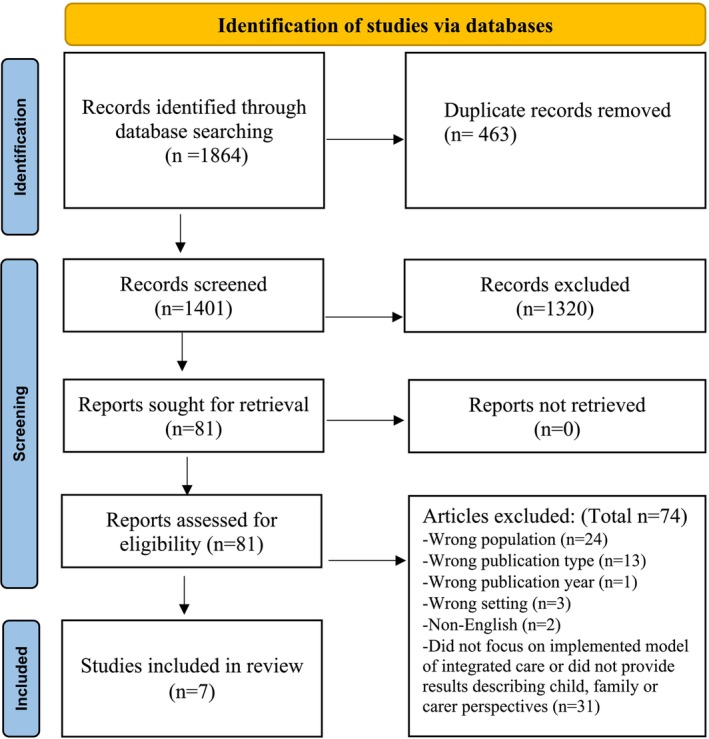
PRISMA flow diagram describing the study identification screening and inclusion process.

A total of 1864 articles were identified from the four database searches. After 463 duplicates were removed, 1401 underwent title and abstract screening. Eighty‐one studies met the inclusion/exclusion criteria and underwent a full‐text review; 74 were excluded because they did not meet criteria, with seven studies retained for data extraction (Figure 1).

### 3.1 Overview of Included Studies

Included were three qualitative studies, three quantitative studies (including one randomised controlled trial) and one mixed methods study (Table 2). Qualitative methods, such as individual semi‐structured interviews or focus groups, were used in three studies. Quantitative measures included the Consumer Assessment of Healthcare Providers and Systems surveys used in two studies (Graham et al. 2017; Looman et al. 2015) to assess parents' healthcare experiences across settings, the Medical Home Family Index and Client Perception of Coordination Questionnaire used in one study (Donnelly et al. 2020), and another study used a quantitative survey of satisfaction developed by that study's authors (Kubendran et al. 2017) (Table 2).

**TABLE 2:** Characteristics of included studies.

Citation and country	Study design	Outcome data collection^a^	Implemented model of integrated care	Setting and lead organisation responsible for delivering PICM	Model of care targets the following types of CMC	Sample details
Kingsnorth et al. (2015 ) **Canada**	Post‐intervention evaluation	Qualitative interviews, focus groups	Key worker service delivery model, where one person acts as a single point of contact and coordinates care across systems and sectors (e.g., healthcare, education and social services) for children and families	A voluntary partnership of three organisations in a local health integration network based in a metropolitan area: Tertiary care children's hospital Children's rehabilitation hospital An organisation responsible for coordinating publicly funded community‐based healthcare	*Not diagnosis specific* Children with at least one chronic condition, complex care needs, medical fragility, and reliance on medical technology according to a definition by Satherley et al. (2021 ): 61% of children had neurological impairment 87% had technological assistance (primarily gastrostomy tube)	12 mothers of CMC aged 6.8 (SD = 5) years
Graham et al. (2017 ) **USA**	Cross‐sectional	Quantitative survey of satisfaction measured using a modified version of the Consumer Assessment Healthcare Providers and Systems survey	Critical Care, Anaesthesia, and Perioperative Extension (CAPE) and Home Ventilation Program Comprehensive, individually tailored care, including 24 h per day/7 days per week access to critical care physicians and other professionals	Based in a metropolitan paediatric hospital, connecting inpatient services, outpatient clinic, rehabilitation programmes, home outreach, school programmes and community services	*Not diagnosis specific* Children with chronic respiratory insufficiency Multiple diagnoses including acquired injury, chronic lung disease, congenital heart disease, dystrophies and spinal muscular atrophy	102 parents (82.4% mothers) of children aged 1–16 years (mean = 6)
Satherley et al. (2021 ) **UK**	Evaluation of user views on key components essential for acceptability, appropriateness and effectiveness	Qualitative interviews	The Children & Young People's Health Partnership Multidisciplinary team: specialist children's nurses who coordinate care across primary, community and hospital settings general practitioners, paediatricians and mental health specialists	Local health system in ethnically and economically diverse inner‐city setting (South London)	*Not diagnosis specific* Long‐term conditions including poorly controlled asthma, chronic constipation, eczema, and epilepsy	19 CMC over 12 years of age 40 mothers (in 3 cases, the father was present with the mother) of CMC aged 1–16 years 8 siblings of CMC; ages of siblings not reported
Looman et al. (2015 ) **USA**	Randomised controlled trial comparing two intervention groups with usual care: One group had access to telephone‐based care coordination only; the other group could access telephone and interactive video‐based coordination. The control group received usual care	Quantitative measures of satisfaction—7 measures from the Consumer Assessment of Healthcare Providers and Systems survey	TeleFamilies service involving advanced practice registered nurses providing high‐intensity telehealth care coordination. Service available during business hours	Special needs clinic serving as a state‐certified medical home within a large general paediatrics clinic Clinic is affiliated with a not‐for‐profit children's hospital in a metropolitan area	*Not diagnosis specific* Applied Cohen's definition of CMC (Cohen et al. 2011). Additionally used three clinical categories to define level of complexity: 50% of children had 3 or more chronic, complex conditions 84% had neurological impairment 47% were dependent on medical technology	148 parents (109 mothers, 8 fathers) or carers (*n* = 24) of CMC aged 2–15 years (mean = 7.1; SD = 4.1) 101 in the intervention arms (50, telephone only; 51, video)
Kubendran et al. (2017 ) **USA**	Observational	Mixed methods, quantitative and qualitative data in a satisfaction survey	Tele‐genetics clinic model to optimise consultation time with geneticist Included initial in‐person consultation with both the paediatrician and genetic counsellor followed by triaged based on complexity to see either clinical geneticist, genetic counsellor or another specialist via tele‐video. Regular case discussion by the team	A major metropolitan hospital with remote access to a clinical geneticist	*Not diagnosis specific* Patients with a non‐syndromic pattern of birth defects, developmental delay, autism, hearing loss, evaluation for Marfan syndrome or neurofibromatosis were prioritised for review	30 parents/carers (gender not provided) of CMC
Cady and Belew (2017 ) **USA**	Evaluation during first year of programme implementation	Qualitative data from focus groups	Patient and family centred model: the Primary Specialty Care Coordination Partnership for Children with Medical Complexity Multidisciplinary team—registered nurse, social worker and appointment coordinator. Coordinating care with ~300 providers in 18 different locations according to family needs	Tertiary urban specialty health system that cares for medically complex children and adults with child‐onset disabling conditions. Includes acute care hospital and 6 outpatient specialty clinics. Links to 14 rural specialty clinics throughout the same state and 4 primary care clinics. One of the 4 clinics is urban‐based and exclusively for non‐English speaking south Asian population—all interactions with families from this clinic require an interpreter. The remaining 3 primary care clinics are rurally located around the state	*Not diagnosis specific* Disabling conditions, including cerebral palsy, spina bifida, muscular dystrophies, epilepsy and congenital syndromes	8 parents (5 mothers; 3 fathers) of 7 CMC aged 0–15 years
Donnelly et al. (2020 ) **USA**	Retrospective pre–post cohort study	Quantitative data using the Medical Home Family Index & Client Perception of Coordination Questionnaire and open questions were collected in a survey written in English and Spanish	The modified Medical Home model. Family‐centred culturally sensitive care coordination provided by pairing an advanced practice nurse (APN) with a Care Coordination Assistant (CCA) across inpatient and outpatient settings	Based in an urban tertiary paediatric hospital, connecting with inpatient, outpatient and community Spanish was the primary language spoken by non‐English‐speaking families The key positions were located in the hospital (APN) and in the community (CCA), Spanish‐speaking CCAs provided logistical support for families	*Not diagnosis specific* Children who had experienced 20 or more days of hospitalisation in the last 2 years and are followed up by three or more specialists: Enrolled children had 4–17 specialists, a mean of 8.6 All children were technology dependent and most required adaptive or mobility equipment	26 parents of CMC aged between 1 and 14 years (mean 5.2, SD 3.5), (96% mothers), 57% Hispanic, 31% White, 8% Black and 4% Asian non‐Hispanic

Abbreviations: CMC, children with medical complexity; PICM, paediatric integrated care model.

^a^ Outcome data collection describes the methods used to collect experiences and perceptions of CMC, parents or carers only.

All seven studies focused on parents or carers of children with a diverse range of medical complexities, for example, neurological impairment, developmental delay, chromosomal anomalies, genetic syndromes and other conditions requiring complex care (Table 2). CMC frequently relied on medical technologies such as nasogastric tube feeding, home ventilation and continuous venous access (Table 2). One study included children with poorly controlled, complex presentations of chronic common conditions such as constipation, asthma and eczema (Satherley et al. 2021). All studies reported on the experiences of parents (Cady and Belew 2017; Donnelly et al. 2020; Graham et al. 2017; Kingsnorth et al. 2015; Kubendran et al. 2017; Looman et al. 2015; Satherley et al. 2021), and one study included experiences of carers (Looman et al. 2015) (Table 2). Only one study reported on the views of CMC and siblings in addition to parents (Satherley et al. 2021). The number of study participants varied from eight in a qualitative study to 148 in the randomised controlled trial. Across the six studies that reported parents' gender/role, of 336 parents, 275 (82%) were mothers.

The results of quality assessment by Hawker's Quality Assessment Tool (Hawker et al. 2002) revealed that five studies were of high quality and two of medium quality (see Table S2).

### 3.2 PICMs and Their Components

The seven studies described seven different PICMs (Table 2). All models emphasised multidisciplinary, team‐based care and aimed to improve the coordination of services for CMC while providing care that was child and family‐focused. PICMs' components included active communication, teamwork and collaboration among all health teams and with families, across all healthcare settings (Cady and Belew 2017; Donnelly et al. 2020; Graham et al. 2017; Kingsnorth et al. 2015; Kubendran et al. 2017; Looman et al. 2015; Satherley et al. 2021). All seven PICMs were not diagnosis‐specific and focused on individual needs while being inclusive of a variety of diagnostic groups of CMC (Table 2).

Appointing one person in the health system to act as a single point of contact and to coordinate care across healthcare teams and with families was a key feature in all seven PICMs (Cady and Belew 2017; Donnelly et al. 2020; Graham et al. 2017; Kingsnorth et al. 2015; Kubendran et al. 2017; Looman et al. 2015; Satherley et al. 2021). Although most PICMs aimed to integrate health services, four also aimed to involve workers from other sectors, for example, education, community care and social services (Donnelly et al. 2020; Graham et al. 2017; Kingsnorth et al. 2015; Satherley et al. 2021). Other features of PICMs included implementing referral pathways and processes of care to improve access to specialist doctors (Cady and Belew 2017; Donnelly et al. 2020; Kubendran et al. 2017; Satherley et al. 2021) improving interdisciplinary teamwork (Cady and Belew 2017; Donnelly et al. 2020; Kingsnorth et al. 2015; Kubendran et al. 2017; Satherley et al. 2021) and providing access to care through telehealth (Kubendran et al. 2017; Looman et al. 2015) (Table 2).

### 3.3 Perceived Benefits and Barriers of PICMs Reported by CMC, Parents and Carers

The aggregated results from seven studies suggest that PICMs are valued by parents and carers of CMC (Table 3). Consistently reported perceived benefits included smoother navigation of health services across providers and sectors; improved communication across teams and with parents (Cady and Belew 2017; Donnelly et al. 2020; Kingsnorth et al. 2015; Kubendran et al. 2017; Looman et al. 2015; Satherley et al. 2021); and simplified and streamlined pathways through care because of a care coordinator acting as a single point of contact (Cady and Belew 2017; Donnelly et al. 2020; Kingsnorth et al. 2015; Satherley et al. 2021). Parents and carers also reported improved experiences and satisfaction with care received through PICMs (Cady and Belew 2017; Donnelly et al. 2020; Kingsnorth et al. 2015; Kubendran et al. 2017; Looman et al. 2015; Satherley et al. 2021). Perceived better access to specialist care (Cady and Belew 2017; Donnelly et al. 2020; Looman et al. 2015), greater understanding among providers of issues faced by families living with CMC (Donnelly et al. 2020; Graham et al. 2017; Looman et al. 2015; Satherley et al. 2021) and improved care coordination across providers (Cady and Belew 2017; Donnelly et al. 2020; Kingsnorth et al. 2015; Satherley et al. 2021) were all frequently reported (Table 3). Family time and cost benefits were reported in only one study (Kingsnorth et al. 2015).

**TABLE 3:** Experiences of benefits of the integrated models of care among CMC, parents or carers as reported in the included papers.

Experiences and impacts reported by CMC parents or carers	Number of studies reporting	Included study citations
CMC, parent, carer satisfaction	7	Cady and Belew (2017 ); Donnelly et al. (2020 ); Graham et al. (2017 ); Kingsnorth et al. (2015 ); Kubendran et al. (2017 ); Looman et al. (2015 ); Satherley et al. (2021 )
Improved care coordination	6	Cady and Belew (2017 ); Donnelly et al. (2020 ); Graham et al. (2017 ); Kingsnorth et al. (2015 ); Looman et al. (2015 ); Satherley et al. (2021 )
Improved communication between providers and family	6	Donnelly et al. (2020 ); Graham et al. (2017 ); Kingsnorth et al. (2015 ); Kubendran et al. (2017 ); Looman et al. (2015 ); Satherley et al. (2021 )
Improved family‐centred care	5	Graham et al. (2017 ); Kingsnorth et al. (2015 ); Kubendran et al. (2017 ); Looman et al. (2015 ); Satherley et al. (2021 )
Smoother navigation via a single point of contact—a care coordinator, key worker or key clinician who knows the child well	5	Graham et al. (2017 ); Kingsnorth et al. (2015 ); Kubendran et al. (2017 ); Looman et al. (2015 ); Satherley et al. (2021 )
Greater sharing of information through improved collaboration among care providers and families	5	Cady and Belew (2017 ); Donnelly et al. (2020 ); Kingsnorth et al. (2015 ); Looman et al. (2015 ); Satherley et al. (2021 )
Improved access to specialist services or doctors	4	Donnelly et al. (2020 ); Kingsnorth et al. (2015 ); Kubendran et al. (2017 ); Satherley et al. (2021 )
Improved self‐management skills	3	Donnelly et al. (2020 ); Kingsnorth et al. (2015 ); Looman et al. (2015 )
Material and emotional support of families	2	Kingsnorth et al. (2015 ); Satherley et al. (2021 )
Greater time and cost effectiveness for families	2	Graham et al. (2017 ); Kubendran et al. (2017 )
Improved trust of healthcare providers	2	Donnelly et al. (2020 ); Satherley et al. (2021 )
Decreased parental burden of care coordination	2	Donnelly et al. (2020 ); Satherley et al. (2021 )
Decreased unmet health needs	1	Satherley et al. (2021 )

Abbreviation: CMC, children with medical complexity.

Care coordinators were identified as essential workers with whom parents developed trusting relationships (Donnelly et al. 2020; Satherley et al. 2021). Parents reported that healthcare providers working within PICMs have an enhanced understanding of family life contexts and the individual needs of CMCs, and this enabled adjustments and tailoring of care to individual needs (Donnelly et al. 2020; Graham et al. 2017; Kingsnorth et al. 2015; Looman et al. 2015; Satherley et al. 2021). Some PICMs recognised significant psychosocial and financial challenges experienced by families of CMC and addressed this by linking families with primary care, social care and education sector supports to build capacity for self‐management (Graham et al. 2017; Kingsnorth et al. 2015; Looman et al. 2015).

Barriers experienced by CMC, parents or carers were less frequently reported (Table 4). Parents in three studies reported that services were underfunded and continued to be fragmented despite a PICM being implemented (Cady and Belew 2017; Kingsnorth et al. 2015; Satherley et al. 2021). In three studies, parents reported not receiving enough information about the new PICM, leading to confusion about model function and scope (Cady and Belew 2017; Kingsnorth et al. 2015; Kubendran et al. 2017). Parents reported a limited understanding of care coordinator roles and how these overlap with the care coordination that parents were already undertaking for their CMC or with coordinators from other services (Cady and Belew 2017; Kingsnorth et al. 2015; Looman et al. 2015; Satherley et al. 2021). Parents in one study highlighted that at times, having a coordinator added complexity, alluding to the need to ‘coordinate the coordinators’ (Cady and Belew 2017). Participants in two studies expressed concerns about the limited ability to share key health information about their child across all involved teams and providers despite PICMs having been implemented (Cady and Belew 2017; Kingsnorth et al. 2015), and this raised issues of care quality and safety (Cady and Belew 2017).

**TABLE 4:** Barriers experienced by CMC^†^, parents or carers as reported in the included papers.

Barriers reported by CMC, parents or carers	Number of studies reporting	Included study citations
Perception that services are underfunded and/or continue to be fragmented	3	Cady and Belew (2017 ); Kingsnorth et al. (2015 ); Satherley et al. (2021 )
Parents/carers not familiar with the model or limited information about the model was shared with families	3	Cady and Belew (2017 ); Kingsnorth et al. (2015 ); Satherley et al. (2021 )
Family must maintain their role as the primary care coordinators to ensure child safety	2	Cady and Belew (2017 ); Kingsnorth et al. (2015 )
Inability to share data across providers and services or limited shared care planning	2	Cady and Belew (2017 ); Kingsnorth et al. (2015 )
Challenges communicating and collaborating to coordinate care across multiple systems, such as primary–secondary and education sectors	2	Cady and Belew (2017 ); Satherley et al. (2021 )
Having multiple coordinators may add to the complexity of care coordination undertaken by the family	1	Looman et al. (2015 )
Minority of parents report poor quality of video/audio during virtual consultations	1	Kubendran et al. (2017 )
Parents uncertain of the role boundaries of the coordinator	1	Cady and Belew (2017 )
Some parents' concerns that not all clinicians have all needed information about their child to provide high quality safe care	1	Cady and Belew (2017 )
Travel for rural families when PICM operates from a metropolitan centre, and there is a lack of expertise in rural regions	1	Cady and Belew (2017 )
Difficulties coordinating care without knowledge of local staff and context	1	Cady and Belew (2017 )

Abbreviation: CMC, children with medical complexity.

## 4 Discussion

This literature review provides a novel synthesis of the experiences of parents, carers and CMC to better understand the benefits and barriers of accessing PICMs.

### 4.1 PICMs Identified in the Review

A wide variety of PICMs were identified, with each of the seven included studies reporting varied model components and purposes. The most common purpose was to deliver care in an integrated and coordinated way, and the involvement of care coordinators and teamwork were key components (Cady and Belew 2017; Donnelly et al. 2020; Graham et al. 2017; Kingsnorth et al. 2015; Kubendran et al. 2017; Looman et al. 2015; Satherley et al. 2021). In recognition of the complex needs of CMC, many of whom needed to access care across different settings, the involvement of workers from hospitals, primary care and community was described (Cady and Belew 2017; Donnelly et al. 2020; Graham et al. 2017; Kingsnorth et al. 2015; Kubendran et al. 2017; Looman et al. 2015; Satherley et al. 2021). Communication, shared care plans and shared health information were also important components of PICMs (Donnelly et al. 2020; Graham et al. 2017; Kingsnorth et al. 2015; Looman et al. 2015). PICMs were place‐based and adjusted to local settings and needs. Overall, PICMs described in the seven included studies were not focused on children with specific diagnoses, although some focused on sub‐groups of children with specific needs, for example, children requiring genetic assessment (Kubendran et al. 2017) or children undergoing surgical procedures (Graham et al. 2017). This diagnosis‐agnostic approach to care provision for CMC has been previously described and is necessary as CMC may have many varied and multiple diagnoses and needs (Breen et al. 2018; Cohen et al. 2011).

Not all children who have a long‐term medical condition require integrated care; however, there is growing agreement that CMC certainly benefit from care integration and coordination, regardless of their specific diagnosis (Altman et al. 2018; Breen et al. 2018; Wolfe et al. 2016; Wolfe et al. 2020). Our review confirms that PICMs tend to be diagnosis‐agnostic, with a focus on addressing complex needs rather than limiting PICMs to specific diagnoses.

### 4.2 Experiences of PICMs and Identified Benefits and Barriers

When caring for CMC, connectivity, team‐based care and care coordination are of particular importance to overcome system fragmentation and to shift away from more traditional siloed single‐disease, single body‐system approaches towards more holistic, team‐based and person‐centred healthcare (Grudniewicz et al. 2018; NHS England 2023; Thistlethwaite 2023; Wolfe et al. 2020). However, several studies reported parent‐ or carer‐perceived barriers to achieving integration in PICMs, including underfunded health and social care services, an overstretched primary care workforce and difficulties engaging with primary care and education sectors (Graham et al. 2017; Kingsnorth et al. 2015). Similar barriers have been observed in other studies (Altman et al. 2018; Breen et al. 2018).

The PICMs described in the included studies mostly originated in large metropolitan children's hospitals, and this continued to limit rural families' access (Cady and Belew 2017). However, one PICM highlighted that although their programme was hospital‐based, all enrolled CMC could access the care coordination service under their PICM, irrespective of where they lived or received their primary care (Donnelly et al. 2020). The benefits of this approach were also described in an Australian study, which reported significant averted travel for families when accessing a PICM that focused on greater collaboration with rural healthcare providers and joint telehealth appointments when appropriate (Breen et al. 2018).

Five studies reported improved access to specialist care, greater collaboration with specialists or sharing of medical records or shared care plans (Donnelly et al. 2020; Kingsnorth et al. 2015; Kubendran et al. 2017; Looman et al. 2015; Satherley et al. 2021). However, in some PICMs, team‐based care was hampered by the inability to share medical records, often because of system factors such as privacy regulations and unlinked electronic medical records systems (Cady and Belew 2017; Kingsnorth et al. 2015; Looman et al. 2015). In one paper, parents/carers reported ongoing frustration with having to repeat the same information about their child to multiple providers, despite an implemented PICM (Cady and Belew 2017). In addition, parents and carers worried that not all healthcare providers have all the information needed to deliver high quality and safe care for their child, because health records may not be routinely shared (Cady and Belew 2017; Kingsnorth et al. 2015). Shared care plans developed in collaboration with CMC and parents and accessible to them have been suggested as a potential solution to ensuring the right and best care is received, but these plans are seldom fully adopted (Cady and Belew 2017; Kingsnorth et al. 2015) or lacked needed functionality (Cady and Belew 2017; Kingsnorth et al. 2015; Satherley et al. 2021). Our results concur with a recent systematic review on the benefits of integrated care, although the limited focus on organisational change to support care integration was also highlighted as a significant systemic barrier (Baxter et al. 2018).

Parents reported a limited understanding of care coordinator roles within PICMs and how these roles integrated with other care coordinators' roles, including the parents' own role as care coordinator for their child (Cady and Belew 2017; Kingsnorth et al. 2015; Looman et al. 2015; Satherley et al. 2021). This created additional complexity for parents and illustrates the need to monitor new models of care for unintended consequences while adjusting to local contextual factors, including taking into account pre‐existing service models. In addition, clear guidance for CMC, parents and carers about the scope, purpose and key roles of PICMs should be provided as the CMC and parents engage with the PICM to manage expectations. The lack of familiarity among parents and variability of the care coordinator role have been confirmed in systematic reviews exploring role effectiveness (Conway et al. 2019) and contextual influences (Hillis et al. 2016). For PICMs to function optimally, a workforce of trained and supported care coordinators/navigators is required. Professionals who take on these key roles need organisational support through clear competency frameworks, co‐designed scopes of practice, role descriptions, professional development specific to their role and opportunities to participate in communities of practice, as outlined in the recent NHS England Workforce Development Framework for Care Coordinators (NHS England 2023). In communities with dominant cultural population groups, it is important that coordinators have the capacity to support engagement with the PICM from a language and cultural perspective (Donnelly et al. 2020).

### 4.3 Knowledge Gaps and Future Implications

Although the perspectives of parents, carers and CMC are paramount to the future success of PICMs, this review highlights a paucity of evidence, with only seven articles meeting our inclusion and exclusion criteria. Only one study included the voices of CMC and of siblings (Satherley et al. 2021), whereas most studies reported the views of parents and almost exclusively mothers. As most PICMs focus on the child and their family, there is a need to explore the views of CMC, fathers and other family members to inform the development, co‐design and evaluation of PICMs in the future.

This review suggests that there are ongoing challenges of engagement among sectors within healthcare (primary care, specialist care and community care) and between healthcare, education and social care. These systemic and structural challenges are difficult to shift (Braithwaite 2018). Implementing electronic health records systems that are connected across health teams, facilities and sectors are needed to support integrated care for CMC. However, a recent scoping review of cross‐organisational access to electronic records demonstrated that this is difficult to achieve in practice (Skeidsvoll Solvang et al. 2025). In addition, co‐designed shared care plans accessible to all professionals involved in care and to CMC families (Wang et al. 2021), in addition to electronic apps and uptake of telehealth, would further support the implementation of PICMs (Asan et al. 2022). The hard work of cross‐sectoral engagement needs to be supported at the system and organisational levels to optimise the value of PICMs. Moreover, understanding potential barriers experienced by CMC and parents/carers and the contextual influences when interacting with PICMs should be carefully considered to inform planning for the implementation and evaluation of PICMs in the future and in shared clinical decision‐making (Albertson et al. 2022; Hillis et al. 2016).

The wide variety of outcome measures across the seven studies limits comparisons of PICMs' effectiveness as perceived by parents and carers. Evidence based on consistently applied and well‐defined outcome measures is needed to better understand the relative benefits of PICMs (Baxter et al. 2018; McLeigh et al. 2022). Patient‐reported experience and outcome measures specifically designed for assessing paediatric care integration, such as the Paediatric Integrated Care Survey (Ziniel et al. 2016), may be more sensitive and appropriate for the evaluation of PICMs and should be routinely used to monitor impacts of PICMs.

### 4.4 Strengths and Limitations

This review begins to address a significant gap in the literature by focusing on the experiences of end‐users of PICMs—parents, carers and CMC. Recognising that CMC often have multiple diagnoses, the literature search concentrated on PICMs developed for CMC rather than on models of care for children with specific diagnoses. This is a considerable strength for identifying literature in line with the definitions of CMC and CMC being cared for in clinical practice (Cohen et al. 2011; Millar et al. 2024). Methodological strengths include the application of the SPIDER framework to guide inclusion and exclusion criteria and using the internationally recognised PRISMA‐ScR recommendations to guide the review and reporting (Tricco et al. 2018). However, the findings of this study should be interpreted in light of some limitations. The interpretation of evidence and implications are limited by the small number of studies available for inclusion. Conducting literature searches on CMC is challenging, given the multitude of different diseases and health conditions that fall under the CMC umbrella, and relevant studies that included CMC and PICMs but used different terminology may have been missed by our search strategy. Additionally, as with all literature reviews, it is possible that we failed to identify some relevant studies. This and the exclusion of grey literature (reports, theses and proceeding) may have limited the comprehensiveness of our review, and these sources should be included in future evidence syntheses. Limiting eligibility to studies published in English and only from high‐income countries may have also limited the transferability of findings to different settings, and the scope should be expanded in future reviews.

### 4.5 Conclusions

The evidence on experiences, benefits and barriers of PICMs for CMC from the perspectives of parents, carers and CMC is scarce. This study provides encouraging evidence of benefits experienced by parents and carers while accessing care for their CMC through PICMs, including smoother system navigation, care coordination, better access to specialist care and high satisfaction levels. Identified barriers highlight the necessity for improved accessible information for parents/carers about the processes, pathways and organisation of PICMs and the roles of care coordinators. The voices of CMC, fathers and siblings are largely absent from the literature. Further research is warranted to inform the co‐design of PICMs that are fit for purpose and meet the needs of CMC and their families.

## Author Contributions

**Yvonne Zurynski:** conceptualization, methodology, writing – review and editing, formal analysis, supervision. **Karen Hutchinson:** conceptualization, methodology, investigation, formal analysis. **Yilin Kang:** conceptualization, methodology, investigation, writing – original draft, data curation. **Maryam Vizheh:** methodology, data curation, writing – review and editing, validation. **Anneliese de Groot:** methodology, validation, data curation, writing – review and editing.

## Ethics Statement

The authors have nothing to report.

## Consent

The authors have nothing to report.

## Conflicts of Interest

The authors declare no conflicts of interest.

## Supporting information


**Table S1.** Example of search strategy in Medline.
**Table S2:** The results of Hawker Quality Assessment Tool (19).

## Data Availability

The data included in this scoping review are publicly available in published peer‐reviewed articles.
