# Supplementary material for: Family Experiences of Integrated Care for Children With Medical Complexity: A Scoping Review
Source: Child Care Health Dev. 2025 May 7;51(3):e70091. doi: 10.1111/cch.70091 (PMC12056461; doi:10.1111/cch.70091)
Supplement: Supplementary file 1 — Table S1. Example of search strategy in Medline. Table S2: The results of Hawker Quality Assessment Tool (19). [file CCH-51-e70091-s001.docx]

**Appendices**

**Table A1.** Example of search strategy in Medline

| 1 | (Child* OR famil* OR caregiver* OR pediatric* OR paediatric* OR teen*) [Title/Abstract] |
| --- | --- |
| 2 | (Attitude* OR view* OR opinion OR experience OR perceptive OR perspective* OR belie* OR “clinical outcome”) [Title/Abstract] |
| 3 | (“Integrated health” OR “integrated healthcare” OR “integrated care” OR “integrated delivery” OR “coordinated care” OR “collaborative care” OR “connected care”) [Title/Abstract] |

**Table A2:** The results of Hawker Tool quality assessment tool (19)

| **Components of assessment** | **Abstract & title** | **Introduction & aims** | **Method & data** | **Sampling** | **Data analysis** | **Ethics & bias** | **Findings/ results** | **Transferability/ generalisability** | **Implications & usefulness** | **Quality score/ Quality rating** |
| --- | --- | --- | --- | --- | --- | --- | --- | --- | --- | --- |
| Kingsnorth et al., 2015 (20) | 4 | 3 | 4 | 4 | 4 | 3 | 4 | 3 | 4 | 33  (High) |
|  |  |  |  |  |  |  |  |  |  |  |
| Graham et al., 2017 (21) | 4 | 4 | 4 | 4 | 4 | 4 | 4 | 3 | 3 | 34  (High) |
| Satherley et al., 2021 (22) | 4 | 4 | 4 | 4 | 4 | 3 | 4 | 4 | 4 | 35  (High) |
| Looman et al., 2015 (23) | 4 | 4 | 4 | 4 | 4 | 1 | 4 | 4 | 4 | 33  (High) |
| Kubendran et al., 2017 (24) | 3 | 3 | 4 | 3 | 3 | 3 | 3 | 3 | 3 | 28  (Medium) |
| Cady & Belew, 2017 (25) | 3 | 4 | 4 | 4 | 4 | 1 | 4 | 4 | 1 | 29  (Medium) |
| Donnelly et al 2020 (26) | 3 | 4 | 4 | 4 | 4 | 1 | 4 | 3 | 4 | 31  (High) |
